# Supplementary material for: Identification of drugs associated with reduced severity of COVID-19 – a case-control study in a large population
Source: eLife. 2021 Jul 27;10:e68165. doi: 10.7554/eLife.68165 (PMC8321549; doi:10.7554/eLife.68165)
Supplement: Transparent reporting form [file elife-68165-transrepform.docx]

***eLife’s* transparent reporting form**

We encourage authors to provide detailed information *within their submission* to facilitate the interpretation and replication of experiments. Authors can upload supporting documentation to indicate the use of appropriate reporting guidelines for health-related research (see [EQUATOR Network](http://www.equator-network.org/%20)), life science research (see the [BioSharing Information Resource](https://biosharing.org/)), or the [ARRIVE guidelines](http://www.plosbiology.org/article/info:doi/10.1371/journal.pbio.1000412) for reporting work involving animal research. Where applicable, authors should refer to any relevant reporting standards documents in this form.

If you have any questions, please consult our Journal Policies and/or contact us: [editorial@elifesciences.org](mailto:editorial@elifesciences.org).

**Sample-size estimation**

- You should state whether an appropriate sample size was computed when the study was being designed
- You should state the statistical method of sample size computation and any required assumptions
- If no explicit power analysis was used, you should describe how you decided what sample (replicate) size (number) to use

Please outline where this information can be found within the submission (e.g., sections or figure legends), or explain why this information doesn’t apply to your submission:

No explicit power calclulation was done. We used all available patients enrolle d in a specified time time interval. Then we assessed how many controls could be tightly matched to each case. For one part of the study most cases could be matched to at least 5 controls and for the other part 2 controls. Then we omitted a small number of cases that did not have sufficient matching controls This is explained in the Methods subsection entitled “Case-control Design and Matching”.

**Replicates**

- You should report how often each experiment was performed
- You should include a definition of biological versus technical replication
- The data obtained should be provided and sufficient information should be provided to indicate the number of independent biological and/or technical replicates
- If you encountered any outliers, you should describe how these were handled
- Criteria for exclusion/inclusion of data should be clearly stated
- High-throughput sequence data should be uploaded before submission, with a private link for reviewers provided (these are available from both GEO and ArrayExpress)

Please outline where this information can be found within the submission (e.g., sections or figure legends), or explain why this information doesn’t apply to your submission:

The issue of replicates does not apply to our case-control study, except as covered by our reply in the previous box. Each case in cohort 1 was matched to five controls. Each case in cohort 2 was matched to 2 controls. In our context, “outliers” were cases for whom there were not five or two suitably matched controls. These outlier cases were dropped from the analysis. This is explained in the Methods subsection entitled “Case-control Design and Matching”.

**Statistical reporting**

- Statistical analysis methods should be described and justified
- Raw data should be presented in figures whenever informative to do so (typically when N per group is less than 10)
- For each experiment, you should identify the statistical tests used, exact values of N, definitions of center, methods of multiple test correction, and dispersion and precision measures (e.g., mean, median, SD, SEM, confidence intervals; and, for the major substantive results, a measure of effect size (e.g., Pearson's r, Cohen's d)
- Report exact p-values wherever possible alongside the summary statistics and 95% confidence intervals. These should be reported for all key questions and not only when the p-value is less than 0.05.

Please outline where this information can be found within the submission (e.g., sections or figure legends), or explain why this information doesn’t apply to your submission:

Statistical analysis is explained in the Methods subsection entitled “Statistical analysis” which we copy here.

## Statistical analysis

Odds ratios for hospitalization for drugs acquired in the case versus control groups and statistical significance were assessed by Fisher's exact test. Correction for multiple testing was performed using the Benjamini-Hochberg procedure (Benjamini and Hochberg, 1995), which gives an estimation of the false discovery rate (FDR) in the list. Statistical analyses were performed in R statistical software version 3.6 (R Foundation for statistical computing).

(For large datasets, or papers with a very large number of statistical tests, you may upload a single table file with tests, Ns, etc., with reference to sections in the manuscript.)

**Group allocation**

- Indicate how samples were allocated into experimental groups (in the case of clinical studies, please specify allocation to treatment method); if randomization was used, please also state if restricted randomization was applied
- Indicate if masking was used during group allocation, data collection and/or data analysis

Please outline where this information can be found within the submission (e.g., sections or figure legends), or explain why this information doesn’t apply to your submission:

There was no group allocation in our study.

**Additional data files (“source data”)**

- We encourage you to upload relevant additional data files, such as numerical data that are represented as a graph in a figure, or as a summary table
- Where provided, these should be in the most useful format, and they can be uploaded as “Source data” files linked to a main figure or table
- Include model definition files including the full list of parameters used
- Include code used for data analysis (e.g., R, MatLab)
- Avoid stating that data files are “available upon request”

Please indicate the figures or tables for which source data files have been provided:

Source data for Figure 1 have been provided as Supplementary File 1. Figure 2 is a schematic diagram summarizing published biochemical knowledge; it is not based on new data.
